# Sustainable Working Life Patterns in a Swedish Twin Cohort: Age-Related Sequences of Sickness Absence, Disability Pension, Unemployment, and Premature Death during Working Life

**DOI:** 10.3390/ijerph191710549

**Published:** 2022-08-24

**Authors:** Annina Ropponen, Pontus Josefsson, Petri Böckerman, Karri Silventoinen, Jurgita Narusyte, Mo Wang, Pia Svedberg

**Affiliations:** 1 Division of Insurance Medicine, Department of Clinical Neuroscience, Karolinska Institutet, 17177 Stockholm, Sweden; 2 Finnish Institute of Occupational Health, 00032 Työterveyslaitos, Finland; 3 IZA Institute of Labor Economics, 53113 Bonn, Germany; 4 School of Business and Economics, University of Jyväskylä, 40014 Jyväskylä, Finland; 5 Labour Institute for Economic Research LABORE, 00100 Helsinki, Finland; 6 Population Research Unit, Faculty of Social Sciences, University of Helsinki, 00014 Helsinki, Finland; 7 Center of Epidemiology and Community Medicine, Stockholm County Council, 104 31 Stockholm, Sweden

**Keywords:** sustainable work, cohort study, sequence analysis, age

## Abstract

We aimed to investigate sustainable working life via age-related sequences of sickness absence (SA), disability pension (DP), unemployment (UE), premature death, and the influence of individual characteristics, accounting for familial confounding. The sample included monozygotic (MZ) and dizygotic (DZ) same-sexed twin pairs with register data (*n* = 47,450) that were followed for 10 years in four age cohorts: 26–35 (*n* = 9892), 36–45 (*n* = 10,620), 46–55 (*n* = 12,964) and 56–65 (*n* = 13,974). A sequence analysis was done in a 7-element state space: 1. “Sustainable working life”: SA/DP 0–30 days and UE 0–90 days; 2. “Unemployment >90 days”: SA/DP 0–30 days and UE > 90 days; 3. “Moderate SA/DP”: SA/DP 30–180 days; 4. “Almost full year of SA/DP”: SA/DP 180–365 days; 5. “Full year of SA/DP”: SA/DP ≥ 365 days; 6. Death; 7. Old-age pension. The largest cluster had a sustainable working life and never experienced states 2–6 (34–59%). Higher education and being married predicted a lower likelihood of experiencing states 2–6. The MZ twin pairs (vs. DZ) were more often in the same cluster suggesting the role of genetic factors. To conclude, the sustainable working life was the largest cluster group. Few individuals had prolonged periods of interruptions of sustainable working life meriting actions, especially in early adulthood for interventions to support workability.

## 1. Introduction

Sustainable working life, in this study is defined as not having interruptions of working life due to various reasons including unemployment, sickness absence (SA), or disability pension (DP) [1]. It is a policy relevant goal due to the increasing need to prolong working careers and the aging population to maintain welfare societies [2,3,4]. Further, the consequences of having either a long or repeated interruptions of working life are large; for example, the SA/DP are linked with increased risk of diseases, reduced well-being, strained economy, poor career development, poor social integration, and premature death [5]. However, although no consensus definition of sustainable working life exists [1,6], there is a need to understand various patterns of working life, with or without interruptions, over the life course. Until now, many earlier studies have focused on SA or DP due to specific diseases or conditions [7,8,9,10], but to the best of our knowledge, population-based cohort studies are rare for working life patterns identified via sequences with a long follow-up over the life course. Hence, a sequence analysis could provide assessment of the sustainable working life over time and informative clusters [11,12].

One of the definitions of sustainable work is “working and living conditions that are such that they support people in engaging and remaining in work throughout an extended working life” [6]. This definition emphasizes the fit between work and the individual characteristics or circumstances during the life course. Regarding life course, it is well-known that age and SA/DP are associated [13], and mental health in early years predicts future participation in working life [14], but they might link with interruptions of working life [15], whereas unemployment in early years of working life may lead to later SA/DP [16]. As mid-life circumstances play a role in interruptions of working life [17], we need further research to identify the interlinkage of various states in working life. It can be hypothesized that the sustainable working life has varying patterns in which SA/DP would increase over time, and unemployment remains stable based on healthy worker effect [18], aging [19] and societal changes related to welfare [20].

The life course approach, that has advanced considerably in recent years due to availability of detailed register data, requires methodological approaches for analyzing time-varying data in longitudinal study designs. These comprehensive datasets require applying time-related and even data-driven statistical techniques to identify patterns in working life. Such a methodology allows reconstructing the course of outcomes over time to identify patterns and detect specific groups following similar development in the outcome [21,22]. Another characteristic with special emphasize is genetics that has been identified to be important for individual differences in SA and DP and being persistent regardless of age [23,24,25]. Because twin studies have shown that many chronic diseases that are also grounds for SA/DP carry a moderate to large genetic influence, e.g., 30% of low back pain [26], 40% depression [27], and 20% anxiety [28], twin studies on sustainable working life would be needed. Twin studies contribute to knowledge based on unrelated individuals as they allow the investigation of the importance of genetic factors for the patterns of a sustainable working life and the associations of other factors. Comparisons of monozygotic (MZ, identical) and dizygotic (DZ, non-identical) twins would allow the assessment of the role of familial confounding in sequences of working life. The interpretation would be based on DZ twin pairs having, on average, 50% of segregating genes in common, whereas MZ twin pairs share 100% genes. Therefore, if MZ co-twins are more similar than DZ co-twins, genetic influences can be assumed. To the best of our knowledge, until now twin studies, with a life course approach to examine sustainable working life, are rare [29,30,31]. Hence, our study contributes to the understanding of longitudinal patterns of sustainable working life and influential factors for the potential identified patterns.

In this study, we aimed to investigate sustainable working life patterns via identification of time-related sequences of sickness absence, disability pension, unemployment, and premature death in a Swedish twin cohort. Furthermore, we aimed to examine the associations between sequences and individual characteristics including zygosity, education, urbanization, and marital status. Finally, we aimed to account for familial confounding via comparison of MZ and same-sexed DZ twin pairs for the sequences.

### 1.1. Sample and Methods

Data from the Swedish Twin Project of Disability Pension and Sickness Absence (STODS) were used including twins identified in the Swedish Twin Registry (STR) and born between 1929 and 1990 (*n* = 119,907 individuals). Data from four national registers covering years 1993 to 2016 were linked to STODS:

- From Karolinska Institutet, the Swedish Twin Register (STR) was used to identify the study population and for background information (zygosity, sex and birthyear).
- From Statistics Sweden, the Longitudinal Integrated Database for Health Insurance and Labor Market Studies (LISA) [32] was used for sociodemographic information (educational level, degree of urbanization, marital status), unemployment and old-age pension.
- From the Swedish Social Insurance Agency, the register Micro Data for Analyses of Social Insurance (MiDAS) was used for information on sickness absence (SA) and disability pension (DP).
- From the Swedish Board of Health and Welfare, the Causes of Death Register was used for dates of death.

The study population with all data included 108,275 twin individuals. The final study sample was limited to MZ and same-sexed DZ twin pairs with documented zygosity [33], with both twins residing in Sweden on December 31 any of the years 1993–2006 when being 25, 35, 45 or 55 years old, and with none of the twins emigrating in the following 10 years (*n* = 47,450). Twins were followed annually for 10 years. Four cohorts were constructed based on the age during follow-up: 26–35 (*n* = 9892), 36–45 (*n* = 10,620), 46–55 (*n* = 12,964) and 56–65 (*n* = 13,974) years. Numbers of complete MZ and DZ same-sex pairs were for 26–35 years old *n* = 2879 and 2067, 36–45 years *n* = 2472 and *n* = 2838, 46–55 years *n* = 2594 and *n* = 3888, and for 56–65 years *n* = 2786 and *n* = 4201, respectively.

### 1.2. Sickness Insurance in Sweden

In Sweden, all individuals aged 16 years or older and in gainful employment or on income benefits, are entitled to sickness benefits from the public sickness insurance system. Only SA spells > 14 days are included. The DP can be granted to residents in Sweden aged 19–64. To be eligible for both SA and DP benefits requires a medically confirmed disorder or injury that affects work capacity. Evaluation of DP includes the assessment of functional capacity, occupational skills, education, work tasks, and work history by the Social Insurance Agency. In Sweden, both SA and DP can be part- or full-time based (25, 50, 75 or 100%) of the ordinary working hours. In the case of part-time SA or DP, net days accounts for that, i.e., two half-days are considered one net day.

### 1.3. Individual Characteristics

The individual characteristics were categorized as: zygosity (MZ; DZ same-sex), educational level (elementary school (≤9 years)/missing; high school (10–12 years); university/collage (>12 years)), degree of urbanization [34] (cities; towns and suburbs; rural areas), and marital status (married; not married). These were measured the year before follow-up started, i.e., at age 25, 35, 45 and 55 respectively.

### 1.4. Statistical Methods

We conducted a longitudinal study with four cohorts of Swedish twins. Each cohort was followed for 10 years. The first cohort was followed between age 26 and 35, the second between age 36 and 45, the third between age 46 and 55 and the fourth between age 56 and 65. To explore patterns of interruptions in working life, we conducted a sequence analysis, one for each cohort [35]. The sequences consisted of 10 yearly observations in a 7-element state space. The states were defined based on SA, DP, unemployment (UE), death and old-age pension (the names for states in italics).

1. *Sustainable working life*: SA/DP 0–30 days and UE 0–90 days
2. *Unemployment >90 days*: SA/DP 0–30 days and UE > 90 days
3. *Moderate SA/DP*: SA/DP 30–180 days
4. *Almost full year of SA/DP*: SA/DP 180–365 days
5. *Full year of SA/DP*: SA/DP ≥ 365 days
6. Death
7. Old-age pension

When assigning states to the individual yearly observations, death was given priority over old-age pension and old-age pension was given priority over SA, DP, and unemployment. First, the state of death was assigned if the individual died during the year or already was dead at the beginning of the year. Then, the state of old-age pension was assigned if the individual had any old-age pension during the year. Last, the remaining states were assigned according to the observed SA, DP, and unemployment. Two states took unemployment into account. These were the states with no more than 30 SA/DP net days, *sustainable working life* and *unemployment*, respectively. The states with more than 30 SA/DP net days did not take unemployment into account, because that would have resulted in states with few observations.

The inter-sequence distances were calculated by the optimal matching algorithm (Needleman–Wunsch), using a substitution matrix created based on observed transitions rates between states. When creating the substitution matrix, the old-age pension state was left out of the calculations. The substitution costs related to the old-age pension state were then set to 0. This makes old-age pension neutral to the other states. The idea of making the old-age pension neutral to the other states was that the old-age pension is an exit from working life, and we wanted to focus on interruptions in working life. Moreover, the calculations of the substitution matrix used all elements of the transition matrix, i.e., the diagonal elements were also included, not just the off-diagonal elements. The insertion or deletion (indel) cost was set to 1.

We then proceeded with a cluster analysis to find clusters of similar individual sequences of interruptions in working life [21]. Four hierarchical agglomerative cluster analyzes were conducted, one for each cohort. The cluster solutions were calculated by the Ward’s linkage method, using the pairwise distance matrix from the sequence analysis. We created six clusters for each cohort. The index plots not grouped by cohort (four age groups with six cluster groups i.e., 24 index plots) were used together with years in states for interpretation of sequences of cluster groups (data not shown but can be obtained from the corresponding author by reasonable request).

Following the cluster analysis, we used conditional logistic regression modeling to analyze the associations between clusters of interruptions in working life and individual characteristics. This applies a discordant twin pair analysis, as twins in a pair with the same value on the dependent variable are dropped because they have no effect on the estimate. As the sample was restricted to same-sexed twins, sex was accounted in these analyses. In essence, the model identifies the associations based on twin differences. Twenty-four conditional logistic regression models were conducted: six for each cohort, one for each cluster within each cohort. The dependent variable was an indicator for belonging to the cluster and the explanatory variables were individual socioeconomic characteristics.

Then the effect of familial confounding factors (i.e., genetics, and shared environment mainly in childhood) on interruptions in working life were assessed. This was done by analyzing if the MZ twin pairs were found more often than same-sex DZ twin pairs in the same cluster of interruptions in working life using the logistic regression model. Four logistic regressions were conducted, one for each cohort. The observations in this analysis were twin pairs, not the individual twins as in the previous analyzes. The dependent variable was an indicator for twins belonging to the same cluster and the explanatory variable was an indicator variable for MZ twins, with the base level being same-sex DZ twins. 

Furthermore, we conducted the zygosity stratified conditional logistic regression analyses (Supplemental material).

All the analyses were performed with the software Stata 17 (Stata Corp, College Station, TX, USA) [36,37].

The ethical vetting was performed and approved by the Regional Ethical Review Board of Stockholm, Sweden (Dnr: 2007/524-31, 2010/1346-32/5, 2014/311-32, 2015/1809-32, 2017/128-32).

## 2. Results

Table 1 shows the characteristics of the four cohorts of this study. The youngest age cohort (26–35 years) had more MZ twins, whereas other cohorts had more DZ twins. The younger cohorts had more often attained higher education levels and were more often living in densely populated areas, compared to the older cohorts. The older cohorts were more often married, compared to the younger cohorts.

The exact group sizes, years in states, and for most frequent sequence orders are presented in Table 2. The respective graphs for each age group (1 to 6) indicating the visualized patterns of sustainable working life are presented in the subsequent figures.

### 2.1. Sustainable Working Life Patterns of Age Cohort 26–35 Years

**Figure 1:**
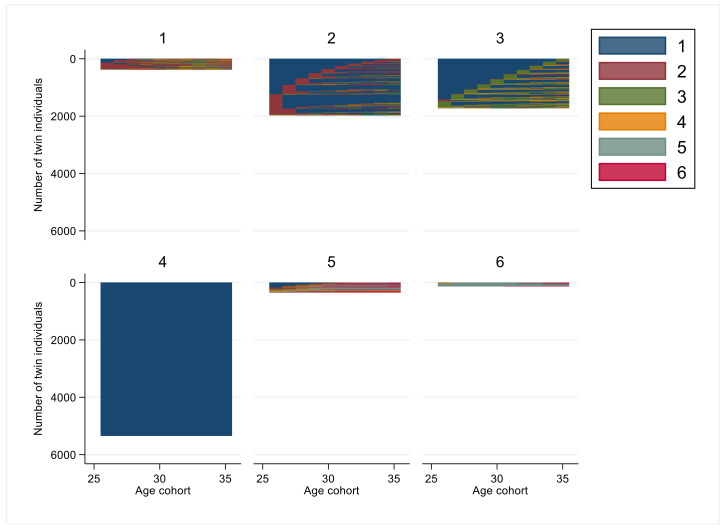
Cluster groups of sequences of interruptions in the working life among the age cohort 26–35 years (Code for colors: 1. *sustainable working life*: SA/DP 0–30 days and UE 0–90 days; 2. *Unemployment > 90 days*: SA/DP 0–30 days and UE > 90 days; 3. *Moderate SA/DP*: SA/DP 30–180 days; 4. *Almost full year of SA/DP*: SA/DP 180–365 days; 5. *Full year of SA/DP*: SA/DP ≥ 365 days; 6. *Death*.

For the age cohort 26–35 years, the six-cluster solution for the age cohort 26–35 years included group 1 (*n* = 377, 4%) characterized with the main states *sustainable working life* and *unemployment > 90 days* (both with mean around 4.7 years) (Table 2, Figure 1). Group 2 (*n* = 1971, 20%) had mainly the state of *sustainable working life* but also were assigned to state of *unemployment > 90 days* (for around two years). Group 3 (*n* = 1726, 17%) had mainly *sustainable working life* combined with almost 1.5 years of *moderate SA/DP*. Group 4 (*n* = 5341, 54%) had *sustainable working life* throughout the study period. Group 5 (*n* = 346, 3%) included *sustainable working life* for almost three years, *moderate SA/DP*, *almost full year SA/DP* and *full year of SA/DP* each for two years. Group 6 (*n* = 131, 1%) had *full year SA/DP* for 8.8 years. Groups 5 and 6 included some who died during the follow-up.

Regarding influential factors for discordant twin pairs in the cluster groups of the youngest age cohort (26–35 years of age), higher education predicted a lower likelihood of belonging to cluster group 2 or 6 and higher likelihood for cluster group 4 (Table 3). Being married was associated with a low likelihood of belonging to cluster groups 1 and 6.

### 2.2. Sustainable Working Life Patterns of the Age Cohort 36–45 Years

**Figure 2:**
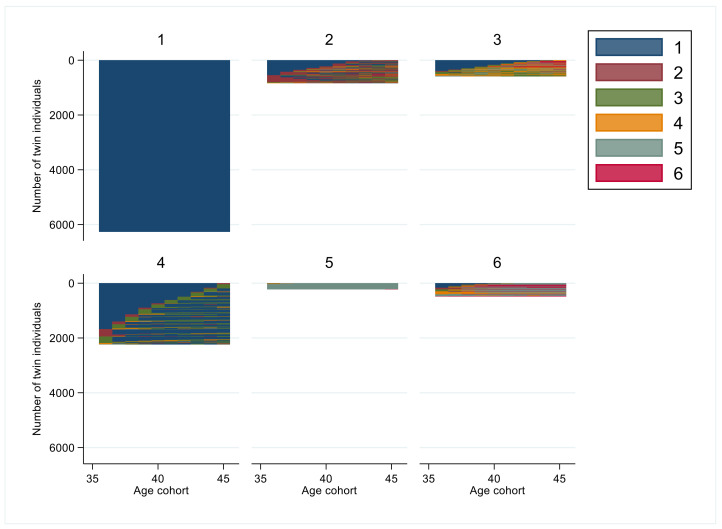
Cluster groups of sequences of interruptions in working life among the age cohort 36–45 years. (Code for colors: 1. *sustainable working life*: SA/DP 0–30 days and UE 0–90 days; 2. *Unemployment > 90 days*: SA/DP 0–30 days and UE > 90 days; 3. *Moderate SA/DP*: SA/DP 30–180 days; 4. *Almost full year of SA/DP*: SA/DP 180–365 days; 5. *Full year of SA/DP*: SA/DP ≥ 365 days; 6. *Death*.

In the age cohort 36–45 years (Figure 2), the six-cluster solution included group 1 (*n* = 6261, 59%) characterized with *sustainable working life* throughout the study period (Table 2). Group 2 (*n* = 836, 8%) had a mean of 6.5 years of *sustainable working life* combined with three years of *unemployment > 90 days*. Group 3 (*n* = 589, 6%) had a mean > 5.5 years of *sustainable working life* and >2 years of *moderate SA/DP* and one year of *almost full year SA/DP*. Group 4 (*n* = 2233, 21%) had a mean of 8.5 years of *sustainable working life* and one year of *moderate SA/DP*. Group 5 (*n* = 220, 2%) was characterized with a *full year SA/DP* during the follow-up. Group 6 (*n* = 481, 5%) had varying SA/DP for most of the follow-up and only 1.7 years of *sustainable working life*. In groups 3 and 6, some individuals died. 

High education predicted a higher likelihood of belonging to cluster groups 1 or 4, and less likelihood of belonging to cluster groups 2, 5 or 6 among 36–45 years old discordant twins. Living in a rural area was associated with a lower likelihood of belonging to cluster group 1 and living in towns and suburbs with a lower likelihood of cluster group 6, whereas being married predicted a lower likelihood for cluster groups 2 or 5 and a higher likelihood for cluster group 1 (Table 3).

### 2.3. Sustainable Working Life Patterns of Age Cohort 46–55 Years

**Figure 3:**
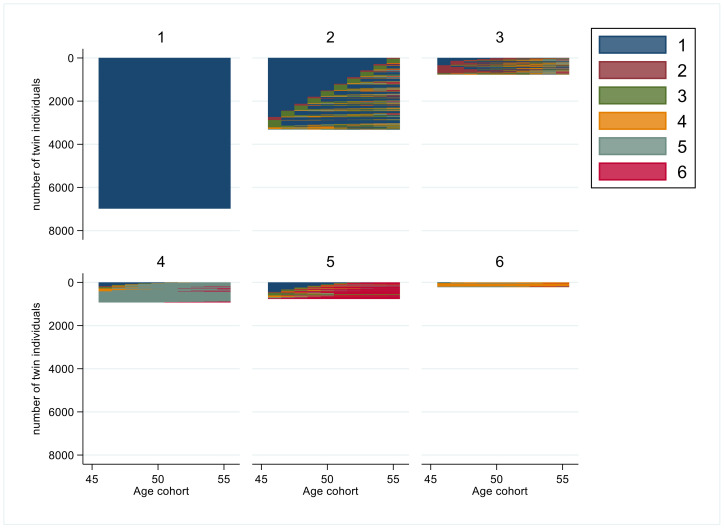
Cluster groups of sequences of interruptions in working life among the age cohort 46–55 years. (Code for colors: 1. *sustainable working life*: SA/DP 0–30 days and UE 0–90 days; 2. *Unemployment > 90 days*: SA/DP 0–30 days and UE > 90 days; 3. *Moderate SA/DP*: SA/DP 30–180 days; 4. *Almost full year of SA/DP*: SA/DP 180–365 days; 5. *Full year of SA/DP*: SA/DP ≥ 365 days; 6. *Death*.

Figure 3 shows a six-cluster solution for the age group 46–55 years. The group 1 (*n* = 6978, 54%) had a *sustainable working life* throughout the study period (Table 2). Group 2 (*n* = 3315, 26%) had a mean of 8 years of *sustainable working life* and 1 year of *moderate SA/DP*. Group 3 (*n* = 768, 6%) had (on average) had 5.7 years of *sustainable working life* combined with 3.5 years of *unemployment > 90 days* and then short periods (≤0.5 years) of *moderate SA/DP* or *almost full year of SA/DP* during the follow-up. Group 4 (*n* = 925, 7%) had a persistent *full year SA/DP* over the years. Group 5 (*n* = 762, 6%) had various lengths of SA/DP for the most of follow-up combined with 2.7 years of *sustainable working life* and 1.7 years of being dead. Group 6 (*n* = 216, 2%) had 9 years of *almost full year SA/DP* during the follow-up.

Among 46–55 years old discordant twin pairs, higher education was associated with a lower likelihood of belonging to cluster groups 3 or 4, but a higher likelihood for cluster group 1. Being married predicted a high likelihood of belonging to cluster group 1 and a lower one to cluster groups 3, 4, or 5 (Table 4). 

### 2.4. Sustainable Working Life Patterns of Age Cohort 56–65 Years

**Figure 4:**
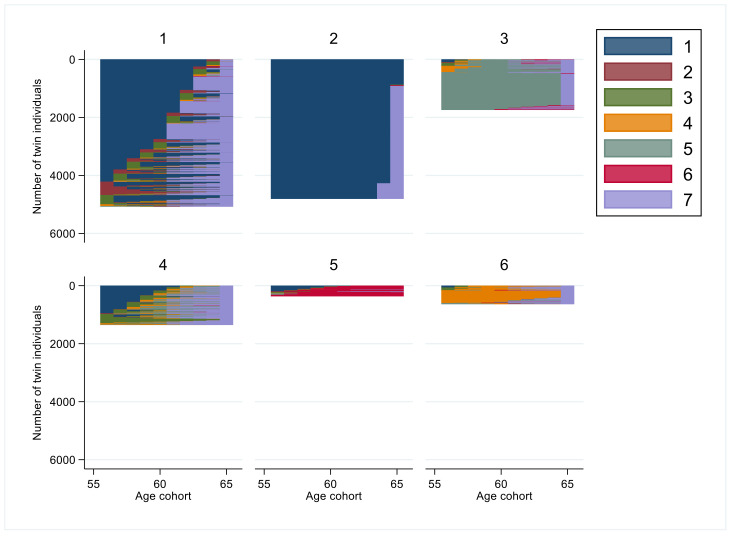
Cluster groups of sequences of interruptions in working life among the age cohort 56–65 years. (Code for colors: 1. *sustainable working life*: SA/DP 0–30 days and UE 0–90 days; 2. *Unemployment > 90 days*: SA/DP 0–30 days and UE > 90 days; 3. *Moderate SA/DP*: SA/DP 30–180 days; 4. *Almost full year of SA/DP*: SA/DP 180–365 days; 5. *Full year of SA/DP*: SA/DP ≥ 365 days; 6. *Death*; and 7. *Old-age pension*).

In the oldest age cohort, 56–65 years olds, the old-age pension became an option as seen in Figure 4. The six clusters included group 1 (*n* = 5076, 36%) with (on average) six years of *sustainable working life* combined with some SA/DP before being on *old-age pension* for two years. In the group 2 (*n* = 4804, 34%) had 9 years of *sustainable working life* before *old-age pension*. Group 3 (*n* = 1740, 12%) had a *full year SA/DP* (mean 8 years) before entering to *old-age pension* or *death*. In group 4 (*n* = 1353, 10%), individuals either had a *sustainable working life* (3 years) or *moderate amount or almost full year of SA/DP* (1–2 years) before being *dead* (7 years). Those in group 5 (*n* = 365, 3%) had two years of *sustainable working life* before being *dead* (7 years). Group 6 (*n* = 636, 5%) included individuals with *almost full year of SA/DP* for 7 years before *old-age pension* (>1 year). 

In the oldest age cohort, higher education predicted a higher likelihood of belonging to cluster group 2, but a lower likelihood for cluster group 3 among discordant twins. Being married was associated with a higher likelihood of belonging to cluster groups 1 or 2 and lower to cluster groups 3 or 5 (Table 4).

### 2.5. Familial Effects on Cluster Membership

In all age cohorts, both members of MZ twin pairs were more often in the same cluster group compared to DZ twin siblings. Among those 26–35 years of age, MZ had an OR of 1.36 (95% CI 1.22, 1.53); for age group 36–45 years, the OR was 1.31 (95%CI 1.18, 1.47), for age group 46–55 years, the OR was 1.34 (95% CI 1.21, 1.48), and for age group 56–65 years the OR was 1.38 (95% CI 1.25, 1.53) compared to DZ pairs. This indicates that genetic effects may affect patterns of interruptions in working life. Furthermore, the analysis of MZ and DZ twins separately (Supplemental Tables S1–S4) indicated that most of the associations between sociodemographic factors and cluster membership retained the magnitude and direction as estimates for all discordant pairs and similar among MZ and DZ twins. This indicates no familial confounding in most of the associations between sociodemographic characteristics and sequence clusters. However, regarding cluster group 5 among the youngest age cohort (26–35 years of age), the associations with educational level were in the opposite direction among MZ twins than for DZ or all twins, pointing towards individual environmental effects. For all four age cohorts, the association between university education and the clusters with only *sustainable working life* (and *old-age pension* for the oldest cohort) seems to be stronger among DZ twins compared to MZ twins. This indicates that the association between university education and *sustainable working life* may be confounded by genetics. The two oldest age groups (46–55 years and 56–65 years) seemed to have individual influences on associations with education in cluster group 6.

## 3. Discussion

This prospective study of Swedish twins assessed working life clusters among four age cohorts over ten years. The aim was to investigate the sustainable working life patterns via identification of age-related sequences of sickness absence, disability pension, unemployment, and premature death. In all four cohorts, the largest cluster had individuals who had a *sustainable working life* and never experienced *unemployment > 90 days*, *moderate SA/DP*, *almost full year of SA/DP, full year of SA/DP* or *death*. In all four cohorts, there were small proportions of individuals with many years with *full year of SA/DP*. Furthermore, the aim was to examine the associations between sequences and individual characteristics. In all cohorts, higher education predicted lower likelihood of experiencing *unemployment > 90 days*, *moderate SA/DP*, *almost full year of SA/DP, full year of SA/DP* or *death*. Being married, except for the youngest cohort (26–35 years), was associated with a lower likelihood of experiencing *unemployment > 90 days*, *moderate SA/DP*, *almost full year of SA/DP, full year of SA/DP* or *death*. Last, we aimed to account for genetic confounding via comparison of MZ and same-sexed DZ twin pairs for the sequences. Being a MZ twin increased the likelihood of belonging to the same cluster group, being indicative of the role of the genetics. However, most associations between sociodemographic characteristics and cluster groups were similar for MZ and DZ twins suggesting no familial confounding. But, for all age cohorts, there were also estimates for MZ that differed from same-sexed DZ twin pairs. The association between university education and *sustainable working life* seemed to be stronger among DZ twins compared to MZ twins. Hence, we cannot rule out genetic influences. To the best of our knowledge, our study is among the first population-based cohort studies to identify working life patterns via sequences with long follow-up over the life course. Our findings are in accordance with the hypothesis that sustainable working life would have varying patterns in which SA and DP would increase over time, while unemployment remains stable over time based on the healthy worker effect [18], aging [19] and societal changes related to welfare [20].

### 3.1. Sustainable Working Life Patterns of Age Cohorts

For the youngest age cohort, 26–35 years of age, the largest sequence group (54%) was those with only *sustainable working life*. The two other main groups had mainly *sustainable working life* in combination with either *unemployment > 90 days* (20%), or *moderate SA/DP* (17%) during follow-up. The other clusters were small (1–4%). This might indicate that those who are most vulnerable to interruptions of working life might not be many but should be identified early to prevent further exclusion from working life. As mental ill-health in early years with or without unemployment is known to predict future participation to working life [14,16], special attention should be paid for absences, interruptions, and health among younger adults experiencing mental ill-health.

In the age cohorts 36–45 years and 46–55 years, the largest group (54–59%) was characterized with a *sustainable working life* throughout the study period. The second largest group (21–26%) had over 8 *years of sustainable working* life but also periods with *moderate SA/DP*. In these age groups, the smallest clusters included 2–8% of individuals with more varying combinations of unemployment, SA/DP, and even death until the end of follow-up. Overall, the finding that most individuals have a sustainable working life is good news. However, the identification of those at risk for absences, interruptions, and health decline, or transfer from temporary absences to permanent ones, should be paid attention at all levels, i.e., at workplaces, communities, and society. Hence these might call for actions regarding working and living conditions and to support people in engaging and remaining in working life [6].

In the oldest age cohort, 56–65 years, the old-age pension became prevalent although the groups with at least rather stable *sustainable working life* combined with or without some SA/DP were the largest (34–36%). Three groups had either a *full year SA/DP* before *death*, or some years of *sustainable working life* or SA/DP before death or old-age-pension (5–12%). Only 3% had a few years of sustainable working life before death. These results are in line with earlier findings that age, and SA/DP are associated [13], and mid-life circumstances play a role in interruptions of working life [17].

### 3.2. Individual Characteristics and Cluster Membership

Regarding the influential factors for belonging to clusters, higher education and being married were associated both with a sustainable working life and with the clusters having interruptions, whereas living area was not. Furthermore, these are the same factors known to be associated with SA/DP, unemployment, and even premature death [1,16], hence higher education and being married might be universally beneficial for sustainable working life. Although we did not find many associations between sociodemographic factors and cluster groups to be influenced by familial/genetic effects, we found clear effects in the two older age groups for associations between higher education and cluster groups. Hence further studies, with even larger sample sizes are needed to confirm the results as we had lower power in some cluster groups when analyzing MZ and DZ twins separately. Hence, the role of genetics on a sustainable working life and any influential factors could be elaborated further.

### 3.3. Strengths and Limitations

Given the nature of this study being longitudinal with a population-based twin cohort with comprehensive national register data, it included several strengths. Our follow-up of age cohorts included no loss to follow-up, no memory or reporting bias and harmonized ten-year follow-ups across ten-year age groups. Furthermore, the twin data enabled us to estimate the role of familial confounding that adds to the knowledge based on general population-based samples. However, a weakness might be the fact that data based on Swedish registers might be less generalizable to other countries than the Nordic ones as they share similar level of welfare and social benefits. Another limitation in this study might be the lack of measurement of various factors, such as working conditions, health behaviors, or other factors [38], that might influence working life patterns. Further studies are needed to fulfill these gaps but also to address potential differences between men and women, occupational groups, or people with ill-health that are enabled to work, such as mental or musculoskeletal disorders or type 2 diabetes.

## 4. Conclusions

Among all age cohorts between 26 and 65 years of age of >47,000 Swedish twins in this prospective study of working life patterns, a sustainable working life was the largest cluster group. The second most common pattern of working life included some interruptions due to unemployment, SA or DP combined with periods of sustainable working life. Familial influences may play a role for a sustainable working life as MZ twin pairs more often belong to the same cluster group than do DZ twins. Only a small group, less than every tenth individual, had prolonged periods of interruptions of sustainable working life meriting actions especially in early adulthood for identification and interventions to support work life participation and sustainable working life.

## Figures and Tables

**Table 1:** Sample characteristics (frequency, *n* and percentage, % of descriptive characteristics) for the study cohorts.

	Study Cohorts (Based on Follow-Up Age Periods)							
	26–35 Years		36–45 Years		46–55 Years		56–65 Years	
	*n*	%	*n*	%	*n*	%	*n*	%
Total (*n* of individuals in the final sample)	9892		10,620		12,964		13,974	
Zygosity								
MZ	5758	58	4944	47	5188	40	5572	40
DZ	4134	42	5676	53	7776	60	8402	60
Sex								
Men	4562	46	5020	47	6370	49	6606	47
Women	5330	54	5600	53	6594	51	7368	53
Education level								
Elementary (≤9 years)	789	8	1073	10	2691	21	4499	32
High school (10–12 years)	5381	54	5940	56	6349	49	6157	44
University/college (>12 years)	3722	38	3607	34	3924	30	3318	24
Degree of urbanization								
Cities (densely populated areas)	4471	45	3943	37	4183	32	4298	31
Towns and suburbs (intermediate density areas)	3785	38	4511	42	5590	43	6302	45
Rural areas (thinly populated areas)	1636	17	2166	20	3191	25	3374	24
Married								
No	9223	93	6079	57	5517	43	5069	36
Yes	669	7	4541	43	7447	57	8905	64

**Table 2:** Frequencies of sequence groups and years in states.

	*n*	%	Years in State							Most Frequent Sequence Order		
			1	2	3	4	5	6	7	Order	*n*	%
**26–35 years of age**												
Cluster group 1	377	4	4.65	4.71	0.47	0.13	0.05	0.00	0.00	2 --> 1 --> 2 --> 1	65	17
Cluster group 2	1971	20	8.11	1.70	0.18	0.02	0.00	0.00	0.00	1 --> 2 --> 1	794	40
Cluster group 3	1726	17	8.08	0.20	1.47	0.21	0.03	0.01	0.00	1 --> 3 --> 1	766	44
Cluster group 4	5341	54	10.00	0.00	0.00	0.00	0.00	0.00	0.00	1	5341	100
Cluster group 5	346	3	2.89	0.77	2.08	2.03	1.92	0.31	0.00	1 --> 4 --> 5	11	3
Cluster group 6	131	1	0.05	0.02	0.21	0.83	8.75	0.15	0.00	5	70	53
Total	9892		8.70	0.58	0.39	0.13	0.19	0.02	0.00			
**36–45 years of age**												
Cluster group 1	6261	59	10.00	0.00	0.00	0.00	0.00	0.00	0.00	1	6261	100
Cluster group 2	836	8	6.53	3.04	0.34	0.07	0.01	0.00	0.00	1 --> 2 --> 1	280	33
Cluster group 3	589	6	5.69	0.38	2.20	1.32	0.29	0.12	0.00	1 --> 4	28	5
Cluster group 4	2233	21	8.48	0.42	1.01	0.08	0.01	0.00	0.00	1 --> 3 --> 1	853	38
Cluster group 5	220	2	0.04	0.01	0.08	0.28	9.60	0.00	0.00	5	158	72
Cluster group 6	481	5	1.66	0.55	1.27	3.39	2.72	0.41	0.00	4	30	6
Total	10,620		8.58	0.37	0.42	0.26	0.34	0.03	0.00			
**46–55 years of age**												
Cluster group 1	6978	54	10.00	0.00	0.00	0.00	0.00	0.00	0.00	1	6978	100
Cluster group 2	3315	26	8.11	0.36	1.19	0.27	0.06	0.01	0.00	1 --> 3 --> 1	1107	33
Cluster group 3	768	6	5.65	3.54	0.53	0.17	0.10	0.01	0.00	1 --> 2 --> 1	141	18
Cluster group 4	925	7	0.64	0.12	0.37	0.72	8.03	0.12	0.00	5	433	47
Cluster group 5	762	6	2.69	0.21	2.65	2.05	0.69	1.70	0.00	1 --> 6	58	8
Cluster group 6	216	2	0.10	0.00	0.32	9.04	0.49	0.05	0.00	4	123	57
Total	12,964		8.00	0.32	0.52	0.40	0.64	0.11	0.00			
**56–65 years of age**												
Cluster group 1	5076	36	6.19	0.76	0.61	0.14	0.06	0.04	2.21	1 --> 7	1392	27
Cluster group 2	4804	34	9.07	0.00	0.00	0.00	0.00	0.00	0.92	1 --> 7	3885	81
Cluster group 3	1740	12	0.07	0.03	0.12	0.36	8.04	0.34	1.04	5 --> 7	1127	65
Cluster group 4	1353	10	3.06	0.12	2.38	1.33	1.63	0.05	1.43	1 --> 4 --> 5 --> 7	148	11
Cluster group 5	365	3	1.98	0.14	0.39	0.22	0.39	6.80	0.08	1 --> 6	105	29
Cluster group 6	636	5	0.12	0.00	0.39	7.08	0.89	0.17	1.35	4 --> 7	294	46
Total	13,974		5.73	0.30	0.49	0.55	1.23	0.25	1.45			

The states were 1. sustainable working life: SA/DP 0–30 days and UE 0–90 days; 2. Unemployment > 90 days: SA/DP 0–30 days and UE > 90 days; 3. Moderate SA/DP: SA/DP 30–180 days; 4. Almost full year of SA/DP: SA/DP 180–365 days; 5. Full year of SA/DP: SA/DP ≥ 365 days; 6. Death; and 7. Old-age pension.

**Table 3:** Conditional odds ratios (OR) with 95% confidence intervals (CI) for associations between sociodemographic factors and sequence clusters among the age cohorts 26–35 years and 36–45 years of age.

	Age Group 26–35 Years											
	Cluster Group 1 (*n* = 634)		Cluster Group 2 (*n* = 2698)		Cluster Group 3 (*n* = 2444)		Cluster Group 4 (*n* = 3674)		Cluster Group 5 (*n* = 548)		Cluster Group 6 (*n* = 190)	
	OR	95% CI	OR	95% CI	OR	95% CI	OR	95% CI	OR	95% CI	OR	95% CI
Education level												
Elementary (≤9 years)	1.00	ref	1.00	ref	1.00	ref	1.00	ref	1.00	ref	1.00	ref
High school (10–12 years)	1.28	0.73, 2.22	**0.66**	**0.46, 0.95**	1.09	0.76, 1.57	**1.66**	**1.18, 2.33**	1.32	0.73, 2.37	**0.06**	**0.01, 0.26**
University/college (>12 years)	0.81	0.37, 1.80	0.80	0.53, 1.22	0.78	0.51, 1.19	**2.34**	**1.60, 3.42**	0.53	0.25, 1.15	**0.00**	**0.00, 0.00**
Degree of urbanization												
Cities (densely populated areas)	1.00	ref	1.00	ref	1.00	ref	1.00	ref	1.00	ref	1.00	ref
Towns and suburbs (intermediate density areas)	0.88	0.50, 1.56	1.18	0.91, 1.52	1.15	0.87, 1.52	0.88	0.70, 1.09	0.86	0.43, 1.72	**0.26**	**0.07, 1.00**
Rural areas (thinly populated areas)	1.80	0.83, 3.91	0.95	0.66, 1.37	0.93	0.65, 1.34	0.93	0.70, 1.25	1.74	0.79, 3.83	0.27	0.01, 6.17
Married												
No	1.00	ref	1.00	ref	1.00	ref	1.00	ref	1.00	ref	1.00	ref
Yes	**0.45**	**0.22, 0.92**	1.19	0.82, 1.71	0.82	0.59, 1.15	1.17	0.86, 1.58	1.41	0.75, 2.64	**0.04**	**0.00, 0.54**
	**Age group 36–45 years**											
	**Cluster Group 1** **(*n* = 4102)**		**Cluster Group 2** **(*n* = 1356)**		**Cluster Group 3** **(*n* = 1058)**		**Cluster Group 4** **(*n* = 3326)**		**Cluster Group 5** **(*n* = 332)**		**Cluster Group 6** **(*n* = 766)**	
Education level												
Elementary (≤9 years)	1.00	ref	1.00	ref	1.00	ref	1.00	ref	1.00	ref	1.00	ref
High school (10–12 years)	1.07	0.82, 1.39	0.84	0.57, 1.24	1.11	0.71, 1.73	**1.35**	**1.02, 1.78**	**0.28**	**0.13, 0.64**	0.85	0.55, 1.32
University/college (>12 years)	**1.85**	**1.36, 2.51**	**0.48**	**0.29, 0.81**	0.99	0.58, 1.71	1.06	0.76, 1.48	**0.14**	**0.05, 0.43**	**0.34**	**0.18, 0.64**
Degree of urbanization												
Cities (densely populated areas)	1.00	ref	1.00	ref	1.00	ref	1.00	ref	1.00	ref	1.00	ref
Towns and suburbs (intermediate density areas)	0.94	0.77, 1.14	1.16	0.80, 1.67	1.08	0.73, 1.60	1.13	0.91, 1.41	1.21	0.58, 2.52	**0.57**	**0.34, 0.94**
Rural areas (thinly populated areas)	**0.69**	**0.54, 0.88**	1.39	0.89, 2.16	1.11	0.69, 1.79	1.27	0.97, 1.67	2.28	0.72, 7.17	0.83	0.48, 1.44
Married												
No	1.00	ref	1.00	ref	1.00	ref	1.00	ref	1.00	ref	1.00	ref
Yes	**1.43**	**1.24, 1.66**	**0.66**	**0.51, 0.86**	0.88	0.67, 1.17	0.87	0.74, 1.02	**0.33**	**0.18, 0.60**	0.98	0.70, 1.39

Estimation method: conditional logistic regression with standard errors adjusted for clustering on twin pairs. The observations are twins. The dependent variable is an indicator for belonging to the cluster. Statistically significant associations in bold. Twins in twin pairs with the same value on the dependent variable are dropped because they have no effect on the estimation.

**Table 4:** Conditional odds ratios (OR) with 95% confidence intervals (CI) for associations between sociodemographic factors and sequence clusters among the age cohorts 46–55 years and 56–65 years of age.

	Age Cohort 46–55 Years											
	Cluster Group 1 (*n* = 5252)		Cluster Group 2 (*n* = 4730)		Cluster group 3 (*n* = 1300)		Cluster Group 4 (*n* = 1402)		Cluster Group 5 (*n* = 1404)		Cluster Group 6 (*n* = 420)	
	OR	95% CI	OR	95% CI	OR	95% CI	OR	95% CI	OR	95% CI	OR	95% CI
Education level												
Elementary (≤9 years)	1.00	ref	1.00	ref	1.00	ref	1.00	ref	1.00	ref	1.00	ref
High school (10–12 years)	1.01	0.84, 1.20	1.17	0.98, 1.41	0.95	0.69, 1.31	**0.69**	**0.51, 0.94**	1.26	0.90, 1.78	0.82	0.44, 1.53
University/college (>12 years)	**1.44**	**1.16, 1.80**	1.14	0.91, 1.44	**0.56**	**0.36, 0.87**	**0.34**	**0.22, 0.53**	1.00	0.64, 1.55	0.46	0.20, 1.07
Degree of urbanization												
Cities (densely populated areas)	1.00	ref	1.00	ref	1.00	ref	1.00	ref	1.00	ref	1.00	ref
Towns and suburbs (intermediate density areas)	1.08	0.91, 1.27	1.05	0.88, 1.26	0.75	0.52, 1.06	0.79	0.57, 1.11	1.02	0.73, 1.43	1.04	0.48, 2.25
Rural areas (thinly populated areas)	0.97	0.78, 1.19	1.14	0.92, 1.42	0.71	0.48, 1.06	0.75	0.51, 1.11	1.07	0.71, 1.60	2.13	0.96, 4.75
Married												
No	1.00	ref	1.00	ref	1.00	ref	1.00	ref	1.00	ref	1.00	ref
Yes	**1.44**	**1.28, 1.63**	1.00	0.88, 1.13	**0.71**	**0.55, 0.92**	**0.60**	**0.47, 0.75**	**0.62**	**0.49, 0.78**	0.91	0.57, 1.43
	**Age cohort 56–65 years**											
	**Cluster Group 1** **(n = 6040)**		**Cluster Group 2** **(n = 5320)**		**Cluster Group 3** **(n = 2456)**		**Cluster Group 4** **(n = 2330)**		**Cluster Group 5** **(n = 686)**		**Cluster Group 6** **(n = 1140)**	
Education level												
Elementary (≤9 years)	1.00	ref	1.00	ref	1.00	ref	1.00	ref	1.00	ref	1.00	ref
High school (10–12 years)	0.97	0.85, 1.12	**1.28**	**1.09, 1.49**	**0.65**	**0.52, 0.79**	0.99	0.78, 1.24	0.86	0.57, 1.30	1.32	0.96, 1.81
University/college (>12 years)	0.90	0.74, 1.09	**1.70**	**1.38, 2.09**	**0.40**	**0.28, 0.57**	1.05	0.76, 1.45	0.53	0.27, 1.07	1.18	0.76, 1.84
Degree of urbanization												
Cities (densely populated areas)	1.00	ref	1.00	ref	1.00	ref	1.00	ref	1.00	ref	1.00	ref
Towns and suburbs (intermediate density areas)	1.01	0.87, 1.17	1.01	0.85, 1.19	1.19	0.92, 1.53	0.81	0.63, 1.04	1.18	0.73, 1.90	0.98	0.67, 1.43
Rural areas (thinly populated areas)	0.91	0.75, 1.09	0.98	0.80, 1.20	1.30	0.97, 1.73	0.90	0.67, 1.21	1.23	0.67, 2.25	1.17	0.76, 1.81
Married												
No	1.00	ref	1.00	ref	1.00	ref	1.00	ref	1.00	ref	1.00	ref
Yes	**1.16**	**1.03, 1.30**	**1.22**	**1.08, 1.39**	**0.58**	**0.48, 0.70**	0.94	0.77, 1.13	**0.50**	**0.36, 0.71**	1.20	0.93, 1.56

Estimation method: conditional logistic regression with standard errors adjusted for clustering on twin pairs. The observations are twins. The dependent variable is an indicator for belonging to the cluster. Statistically significant associations in bold. Twins in twin pairs with the same value on the dependent variable are dropped because they have no effect on the estimation.

## Data Availability

The data that support the findings of this study are available from the original sources: the Swedish Twin Registry, Statistics Sweden, Swedish Social Insurance Agency and the Swedish National Board of Health and Welfare. Restrictions apply to the availability of the data used in this study based on the Swedish Twin project Of Disability pension and Sickness absence (STODS), which were used with ethical permission for the current study and therefore are not publicly available.

## Supplementary Materials

The following supporting information can be downloaded at: https://www.mdpi.com/article/10.3390/ijerph191710549/s1. Table S1 Conditional odds ratios (OR) with 95% confidence intervals (CI) for associations between sociodemographic factors and sequence clusters among monozygotic (MZ) and dizygotic (DZ) twins in the age cohort 26–35 years. Table S2 Conditional odds ratios (OR) with 95% confidence intervals (CI) for associations between sociodemographic factors and sequence clusters among monozygotic (MZ) and dizygotic (DZ) twins in the age cohort 36–45 years. Table S3 Conditional odds ratios (OR) with 95% confidence intervals (CI) for associations between sociodemographic factors and sequence clusters among monozygotic (MZ) and dizygotic (DZ) twins in the age cohort 46–55 years. Table S4 Conditional odds ratios (OR) with 95% confidence intervals (CI) for associations between sociodemographic factors and sequence clusters among monozygotic (MZ) and dizygotic (DZ) twins in the age cohort 56–65 years.
