# Supplementary material for: Sustainable Working Life Patterns in a Swedish Twin Cohort: Age-Related Sequences of Sickness Absence, Disability Pension, Unemployment, and Premature Death during Working Life
Source: Int J Environ Res Public Health. 2022 Aug 24;19(17):10549. doi: 10.3390/ijerph191710549 (PMC9517844; doi:10.3390/ijerph191710549)
Supplement: Supplementary file 1 [file ijerph-19-10549-s001.zip › ijerph-1803698-supplementary.pdf]

Table S1. Conditional odds ratios (OR) with 95% confidence intervals (CI) for associations between sociodemographic factors and sequence clusters among monozygotic (MZ) and dizygotic (DZ) twins in the age cohort 26-35 years.

	**26-35 years old MZ twins**											
	**Cluster group 1 (*n* =310)**		**Cluster group 2 (*n* = 1504)**		**Cluster group 3 (*n* =1396)**		**Cluster group 4 (*n* = 1990)**		**Cluster group 5 (*n* = 282)**		**Cluster group 6 (*n* = 78)**	
	**OR**	**95% CI**	**OR**	**95% CI**	**OR**	**95% CI**	**OR**	**95% CI**	**OR**	**95% CI**	**OR**	**95% CI**
Education level												
Elementary (≤9 years)	1.00	ref	1.00	ref	1.00	ref	1.00	ref	1.00	ref	1.00	ref
High school (10-12 years)	1.13	0.43, 2.97	0.64	0.34, 1.22	1.36	0.77, 2.40	1.41	0.83, 2.41	0.56	0.19, 1.67	na	-
University/college (>12 years)	1.12	0.26, 4.90	0.74	0.35, 1.54	1.02	0.53, 1.99	1.74	0.93, 3.25	0.52	0.12, 2.15	na	-
Degree of urbanization												
Cities (densely populated areas)	1.00	ref	1.00	ref	1.00	ref	1.00	ref	1.00	ref	1.00	ref
Towns and suburbs (intermediate density areas)	1.08	0.45, 2.59	1.37	0.96, 1.95	0.98	0.67, 1.45	0.82	0.60, 1.13	1.15	0.41, 3.23	0.20	0.02, 1.76
Rural areas (thinly populated areas)	**6.55**	**1.20, 35.73**	0.75	0.44, 1.29	1.01	0.61, 1.69	0.87	0.56, 1.33	2.75	0.77, 9.89	0.00	0.00, 0.00
Married												
No	1.00	ref	1.00	ref	1.00	ref	1.00	ref	1.00	ref	1.00	ref
Yes	0.45	0.15, 1.34	1.10	0.61, 2.01	0.84	0.53, 1.32	1.25	0.79, 1.98	1.17	0.52, 2.63	na	-

	**26-35 years old DZ twins**											
	**Cluster group 1 (*n* = 324)**		**Cluster group 2 (*n* = 1194)**		**Cluster group 3 (*n* = 1048)**		**Cluster group 4 (*n* = 1684)**		**Cluster group 5 (*n* = 266)**		**Cluster group 6 (*n* = 112)**	
Education level												
Elementary (≤9 years)	1.00	ref	1.00	ref	1.00	ref	1.00	ref	1.00	ref	1.00	ref
High school (10-12 years)	1.40	0.71, 2.73	0.68	0.44, 1.06	0.91	0.55, 1.48	**1.83**	**1.17, 2.84**	2.13	0.98, 4.64	**0.06**	**0.01, 0.60**
University/college (>12 years)	0.70	0.27, 1.85	0.87	0.52, 1.43	0.62	0.35, 1.10	**2.82**	**1.74, 4.57**	0.41	0.15, 1.17	na	-
Degree of urbanization												
Cities (densely populated areas)	1.00	ref	1.00	ref	1.00	ref	1.00	ref	1.00	ref	1.00	ref
Towns and suburbs (intermediate density areas)	0.74	0.34, 1.63	1.02	0.71, 1.49	1.40	0.93, 2.10	0.95	0.70, 1.29	0.61	0.23, 1.59	0.52	0.09, 3.09
Rural areas (thinly populated areas)	0.93	0.36, 2.45	1.12	0.67, 1.87	0.86	0.52, 1.44	1.02	0.69, 1.51	1.18	0.40, 3.41	1.11	0.10, 12.85
Married												
No	1.00	ref	1.00	ref	1.00	ref	1.00	ref	1.00	ref	1.00	ref
Yes	0.50	0.19, 1.29	1.25	0.79, 1.98	0.82	0.50, 1.34	1.08	0.72, 1.63	1.77	0.65, 4.80	na	-

na = not able to be assessed due to low number of individuals.

Table S2. Conditional odds ratios (OR) with 95% confidence intervals (CI) for associations between sociodemographic factors and sequence clusters among monozygotic (MZ) and dizygotic (DZ) twins in the age cohort 36-45 years.

	**36-45 years old MZ twins**											
	**Cluster group 1 (*n* = 1740)**		**Cluster group 2 (*n* = 590)**		**Cluster group 3 (*n* = 484)**		**Cluster group 4 (*n* = 1478)**		**Cluster group 5 (*n* = 130)**		**Cluster group 6 (*n* = 310)**	
	**OR**	**95% CI**	**OR**	**95% CI**	**OR**	**95% CI**	**OR**	**95% CI**	**OR**	**95% CI**	**OR**	**95% CI**
Education level												
Elementary (≤9 years)	1.00	ref	1.00	ref	1.00	ref	1.00	ref	1.00	ref	1.00	ref
High school (10-12 years)	0.90	0.57, 1.43	0.52	0.23, 1.18	0.92	0.45, 1.88	1.47	0.91, 2.37	0.56	0.17, 1.89	1.14	0.54, 2.40
University/college (>12 years)	1.09	0.64, 1.87	**0.28**	**0.10, 0.81**	0.96	0.39, 2.38	1.54	0.87, 2.71	0.56	0.11, 2.93	0.46	0.12, 1.72
Degree of urbanization												
Cities (densely populated areas)	1.00	ref	1.00	ref	1.00	ref	1.00	ref	1.00	ref	1.00	ref
Towns and suburbs (intermediate density areas)	0.97	0.72, 1.32	1.62	0.91, 2.88	0.92	0.51, 1.66	1.03	0.73, 1.46	1.61	0.45, 5.79	**0.39**	**0.16, 0.98**
Rural areas (thinly populated areas)	0.76	0.52, 1.13	1.42	0.70, 2.87	1.01	0.49, 2.06	1.22	0.78, 1.90	2.22	0.40, 12.45	0.52	0.21, 1.27
Married												
No	1.00	ref	1.00	ref	1.00	ref	1.00	ref	1.00	ref	1.00	ref
Yes	**1.47**	**1.17, 1.84**	0.71	0.48, 1.06	1.12	0.73, 1.72	**0.75**	**0.59, 0.96**	0.34	0.12, 0.93	1.05	0.55, 1.99
	**36-45 years old DZ twins**											
	**Cluster group 1 (*n* = 2362)**		**Cluster group 2 (*n* = 766)**		**Cluster group 3 (*n* = 574)**		**Cluster group 4 (*n* = 1848)**		**Cluster group 5 (*n* = 202)**		**Cluster group 6 (*n* = 456)**	
Education level												
Elementary (≤9 years)	1.00	ref	1.00	ref	1.00	ref	1.00	ref	1.00	ref	1.00	ref
High school (10-12 years)	1.15	0.84, 1.59	1.01	0.65, 1.58	1.28	0.71, 2.29	1.29	0.91, 1.82	**0.16**	**0.05, 0.56**	0.75	0.43, 1.29
University/college (>12 years)	**2.43**	**1.66, 3.55**	0.58	0.32, 1.04	1.02	0.51, 2.06	0.87	0.58, 1.31	**0.06**	**0.01, 0.29**	**0.30**	**0.14, 0.64**
Degree of urbanization												
Cities (densely populated areas)	1.00	ref	1.00	ref	1.00	ref	1.00	ref	1.00	ref	1.00	ref
Towns and suburbs (intermediate density areas)	0.92	0.71, 1.19	0.91	0.56, 1.49	1.25	0.73, 2.15	1.18	0.89, 1.56	1.21	0.47, 3.10	0.67	0.36, 1.25
Rural areas (thinly populated areas)	**0.63**	**0.46, 0.88**	1.35	0.75, 2.42	1.16	0.61, 2.22	1.31	0.92, 1.86	2.82	0.61, 12.98	1.07	0.53, 2.15
Married												
No	1.00	ref	1.00	ref	1.00	ref	1.00	ref	1.00	ref	1.00	ref
Yes	**1.42**	**1.17, 1.71**	**0.61**	**0.43, 0.87**	0.72	0.49, 1.05	0.97	0.79, 1.19	**0.35**	**0.16, 0.77**	0.98	0.65, 1.47

Table S3. Conditional odds ratios (OR) with 95% confidence intervals (CI) for associations between sociodemographic factors and sequence clusters among monozygotic (MZ) and dizygotic (DZ) twins in the age cohort 46-55 years.

	**46-55 years old MZ twins**											
	**Cluster group 1 (*n* = 1896)**		**Cluster group 2 (*n* = 1800)**		**Cluster group 3 (*n* = 480)**		**Cluster group 4 (*n* = 484)**		**Cluster group 5 (*n* = 526)**		**Cluster group 6 (*n* = 170)**	
	**OR**	**95% CI**	**OR**	**95% CI**	**OR**	**95% CI**	**OR**	**95% CI**	**OR**	**95% CI**	**OR**	**95% CI**
Education level												
Elementary (≤9 years)	1.00	ref	1.00	ref	1.00	ref	1.00	ref	1.00	ref	1.00	ref
High school (10-12 years)	0.87	0.62, 1.21	1.33	0.94, 1.86	0.68	0.33, 1.39	0.62	0.35, 1.09	1.46	0.77, 2.79	1.57	0.54, 4.54
University/college (>12 years)	0.96	0.63, 1.47	1.47	0.94, 2.29	**0.37**	**0.14, 0.98**	0.48	0.20, 1.14	1.10	0.48, 2.55	2.30	0.49, 10.67
Degree of urbanization												
Cities (densely populated areas)	1.00	ref	1.00	ref	1.00	ref	1.00	ref	1.00	ref	1.00	ref
Towns and suburbs (intermediate density areas)	1.17	0.88, 1.55	0.85	0.63, 1.15	0.88	0.49, 1.57	0.80	0.44, 1.46	1.11	0.64, 1.94	1.02	0.34, 3.12
Rural areas (thinly populated areas)	0.73	0.50, 1.08	1.21	0.82, 1.79	0.82	0.41, 1.67	0.92	0.46, 1.86	1.20	0.61, 2.34	2.62	0.87, 7.83
Married												
No	1.00	ref	1.00	ref	1.00	ref	1.00	ref	1.00	ref	1.00	ref
Yes	1.20	0.97, 1.49	0.94	0.75, 1.17	0.87	0.57, 1.33	0.79	0.51, 1.24	0.85	0.56, 1.28	1.24	0.57, 2.70
	**46-55 years old DZ twins**											
	**Cluster group 1 (*n* = 3356)**		**Cluster group 2 (*n* = 2930)**		**Cluster group 3 (*n* = 820)**		**Cluster group 4 (*n* = 918)**		**Cluster group 5 (*n* = 878)**		**Cluster group 6 (*n* = 250)**	
Education level												
Elementary (≤9 years)	1.00	ref	1.00	ref	1.00	ref	1.00	ref	1.00	ref	1.00	ref
High school (10-12 years)	1.06	0.86, 1.31	1.12	0.90, 1.39	1.05	0.73, 1.52	0.76	0.53, 1.10	1.19	0.79, 1.80	0.64	0.28, 1.47
University/college (>12 years)	1.68	1.29, 2.18	1.03	0.79, 1.35	0.64	0.38, 1.06	**0.31**	**0.18, 0.52**	0.97	0.57, 1.64	**0.22**	**0.07, 0.68**
Degree of urbanization												
Cities (densely populated areas)	1.00	ref	1.00	ref	1.00	ref	1.00	ref	1.00	ref	1.00	ref
Towns and suburbs (intermediate density areas)	1.04	0.84, 1.29	1.21	0.96, 1.52	0.65	0.42, 1.03	0.82	0.55, 1.24	0.94	0.61, 1.45	1.24	0.44, 3.53
Rural areas (thinly populated areas)	1.08	0.84, 1.39	1.14	0.88, 1.49	0.65	0.40, 1.05	0.69	0.43, 1.10	0.97	0.58, 1.63	2.36	0.75, 7.39
Married												
No	1.00	ref	1.00	ref	1.00	ref	1.00	ref	1.00	ref	1.00	ref
Yes	**1.57**	**1.36, 1.82**	1.03	0.88, 1.20	**0.64**	**0.46, 0.88**	**0.53**	**0.40, 0.70**	**0.54**	**0.40, 0.71**	0.72	0.40, 1.30

Table S4. Conditional odds ratios (OR) with 95% confidence intervals (CI) for associations between sociodemographic factors and sequence clusters among monozygotic (MZ) and dizygotic (DZ) twins in the age cohort 56-65 years.

	**56-65 years old MZ twins**											
	**Cluster group 1 (*n* = 2284)**		**Cluster group 2 (*n* = 1944)**		**Cluster group 3 (*n* = 828)**		**Cluster group 4 (*n* = 926)**		**Cluster group 5 (*n* = 242)**		**Cluster group 6 (*n* = 440)**	
	**OR**	**95% CI**	**OR**	**95% CI**	**OR**	**95% CI**	**OR**	**95% CI**	**OR**	**95% CI**	**OR**	**95% CI**
Education level												
Elementary (≤9 years)	1.00	ref	1.00	ref	1.00	ref	1.00	ref	1.00	ref	1.00	ref
High school (10-12 years)	0.77	0.59, 1.01	1.16	0.87, 1.55	0.74	0.49, 1.11	1.51	0.99, 2.31	0.81	0.39, 1.69	**1.91**	**1.02, 3.56**
University/college (>12 years)	0.72	0.50, 1.03	1.12	0.76, 1.65	0.62	0.31, 1.25	**2.10**	**1.15, 3.85**	0.55	0.15, 1.99	**2.45**	**1.08, 5.53**
Degree of urbanization												
Cities (densely populated areas)	1.00	ref	1.00	ref	1.00	ref	1.00	ref	1.00	ref	1.00	ref
Towns and suburbs (intermediate density areas)	1.11	0.86, 1.44	0.94	0.71, 1.23	1.38	0.87, 2.20	0.67	0.44, 1.04	0.92	0.40, 2.12	1.01	0.55, 1.85
Rural areas (thinly populated areas)	0.96	0.69, 1.34	0.89	0.62, 1.27	1.67	0.97, 2.87	0.62	0.36, 1.08	1.19	0.38, 3.75	1.70	0.73, 3.93
Married												
No	1.00	ref	1.00	ref	1.00	ref	1.00	ref	1.00	ref	1.00	ref
Yes	1.16	0.96, 1.42	0.94	0.76, 1.17	0.78	0.57, 1.07	0.98	0.71, 1.35	0.75	0.43, 1.29	1.23	0.79, 1.91
	**56-65 years old DZ twins**											
	**Cluster group 1 (*n* = 3756)**		**Cluster group 2 (*n* = 3764)**		**Cluster group 3 (*n* = 1628)**		**Cluster group 4 (*n* = 1404)**		**Cluster group 5 (*n* = 444)**		**Cluster group 6 (*n* = 700)**	
Education level												
Elementary (≤9 years)	1.00	ref	1.00	ref	1.00	ref	1.00	ref	1.00	ref	1.00	ref
High school (10-12 years)	1.06	0.90, 1.26	**1.32**	**1.10, 1.58**	**0.62**	**0.48, 0.79**	0.83	0.62, 1.09	0.95	0.57, 1.57	1.17	0.81, 1.69
University/college (>12 years)	0.97	0.77, 1.21	**2.02**	**1.57, 2.60**	**0.34**	**0.22, 0.51**	0.80	0.54, 1.18	0.58	0.25, 1.34	0.86	0.50, 1.46
Degree of urbanization												
Cities (densely populated areas)	1.00	ref	1.00	ref	1.00	ref	1.00	ref	1.00	ref	1.00	ref
Towns and suburbs (intermediate density areas)	0.96	0.79, 1.16	1.04	0.84, 1.28	1.11	0.82, 1.51	0.89	0.64, 1.23	1.40	0.75, 2.60	0.95	0.59, 1.54
Rural areas (thinly populated areas)	0.88	0.70, 1.10	1.03	0.80, 1.31	1.16	0.82, 1.64	1.03	0.72, 1.49	1.27	0.61, 2.67	0.97	0.57, 1.63
Married												
No	1.00	ref	1.00	ref	1.00	ref	1.00	ref	1.00	ref	1.00	ref
Yes	**1.16**	**1.00, 1.34**	**1.40**	**1.19, 1.63**	**0.50**	**0.40, 0.63**	0.92	0.73, 1.17	**0.37**	**0.23, 0.59**	1.20	0.87, 1.66
